# Evaluation of the Potential of Fungal Biopesticides for the Biological Control of the Seed Bug, *Elasmolomus pallens* (Dallas) (Hemiptera: Rhyparochromidae)

**DOI:** 10.3390/insects11050277

**Published:** 2020-05-01

**Authors:** Fredrick Fidelis Umaru, Khanom Simarani

**Affiliations:** 1 Institute of Biological Sciences, Faculty of Science, University of Malaya, Kuala Lumpur 50603, Malaysia; umarufred@gmail.com; 2 Department of Biological Sciences, Faculty of Science, Taraba State University, Jalingo 660243, Nigeria

**Keywords:** biological control, aflatoxin, *Aspergillus flavus*, *Metarhizium anisopliae*, insect cadaver, biocontrol agent, groundnut

## Abstract

*Elasmolomus pallens* is a post-harvest insect pest of groundnuts which causes severe yield loss to farmers, particularly in Africa and Asia. Resistance to synthetic chemicals has been on the rise among insects and is a constraint on insecticides regulations. In view of the drive for alternative approaches to synthetic insecticides, this study evaluated the potential of biopesticides based on entomopathogenic fungi against *E. pallens* under laboratory conditions. Fungal isolates from the bug cadaver including *Fusarium proliferatum* F1, *Aspergillus tamarii* F2, *A. flavus* F3, *Trichoderma atroviride* F4, *A. niger* F4, and *Metarhizium anisopliae* (Meschn.) Sorokin, originating from the cadaver of *Zonocerus variegatus* were screened for virulence against the bug. Adult bugs were dipped briefly in conidial concentration 1 × 10^8^ conidial mL^−1^ and observed at 25 ± 2 °C, 80 ± 10 RH and 14: 10 L:D for 10 days. The fungal isolates caused mortality ranging from 48 to 100% based on their potential to infect and kill the bug. Five conidial concentrations (1 × 10^4^ to 1 × 10^8^ conidia mL^−1^) were evaluated against adult bugs in the multiple-dose virulence bioassay. Lethal concentrations (LC_50_ and LC_90_) values of 6.75 × 10^6^ and 4.42 × 10^9^ conidia mL^−1^ were obtained for *A. flavus* F3 while *M. anisopliae* had 8.0 × 10^6^ and 6.14 × 10^8^ conidia mL^−1^ respectively. Lethal time (LT_50_ and LT_90_) values were 3.3 and 6.2 days for *A. flavus* F3 compared to 3.6 and 5.6 days for *M. anisopliae*, respectively. Thus, *A. flavus* F3 showed potential against *E. pallens*; and can be considered as an ideal isolate for incorporation into formulations for field applications.

## 1. Introduction

*Elasmolomus pallens* (Dallas) is a seed bug which belongs to the family Rhyparochromidae, within the superfamily Lygaeoidea and in the order Hemiptera. The family was formerly considered as a subfamily within the Lygaeidae until the recent reanalysis of the phylogenetic relationship of the taxon [1]. Members of the family are cosmopolitan in nature and are considered the largest of the lygaeoid bugs [2]. *E. pallens*, formerly known as *E. sordidus* (Fabricius, 1787), has a wider distribution cutting across tropical, subtropical, and parts of the temperate regions of Africa, Asia, and the Pacific Islands [3]. An important determinant in the dispersal of *E. pallens* is its association with the legume, *Arachis hypogea* L., also called peanuts or groundnuts [4]. 

In Sub-Saharan African countries where peanut farming is practiced and processing implements are lacking, harvested peanuts are left for weeks on the field to dry before stripping. When this happens, the bugs congregate beneath the heaps and destroy the kernels contained inside intact pods by using their rostrum to suck out the oil from the kernels. The attacked kernels become soft, oilier and shriveled [5]. 

In Asia, post-harvest losses of groundnuts due to insect infestation have ranged from 10–25% of the production, resulting in direct losses, while indirect losses due to poor quality of the produce impacts its trade and use [6]. In India, Dabhade and Bapodra [7] have reported 48.57% post-harvest yield loss of groundnuts due to insect infestation. While in Nigeria, Samaila and Malgwi [8] described a sister seed bug *R. littoralis* as one of the major insect pests of groundnuts which causes 68% post-harvest yield loss of the crop per year. 

Since *E. pallens* is a pest of serious economic concern to agricultural production and global trade, the need for its control becomes imperative. However, commonly used methods of control have been the use of *Cephalonomia* (a bethylid wasp) to parasitize the eggs of *E. pallens*, while the reduviid *Coranus pallidus* is applied as a predator on adults and nymphs. Surface layering and dusting of peanut stacks with 0.5% lindane and 2% malathion have also been found to be effective for the control of the bug [4]. But whether these insect parasites used for the control of *E. pallens* will not assume pest status remains cautionary, and likewise, the impact of synthetic chemicals on biodiversity and health. The caution is necessary because synthetic insecticides, such as the commonly used organophosphates, pose a deleterious impact on both humans and the environment [9]. Considering all the potent dangers, environment-friendly approaches to insect pest control via alternative natural pesticides are needed. This position is corroborated by Pretty and Bharucha [10], that alternative pest control methods, instead of synthetic pesticides should be adopted to mitigate pest damage with less cost and minimal negative outcomes.

Entomopathogenic fungi (EPF) are organisms in nature that specialize in killing insects by penetrating their cuticles into the haemocoel, unlike other pathogens that must be ingested before infection is initiated [11]. They are widespread, common regulators of the insect population under epizootic conditions [12,13], and producers of unique insecticidal compounds [14]. The continued efforts of investigating the occurrence and hosts associated distributions of EPF is essential to establishing their suitability and application in appropriate locations, since the degree of virulence among strains could be attributed to genetic variations and geographic locations [15]. According to Jaronski [16], mycopesticides developed in some countries may fail in terms of efficacy in another country due to the factors of strain and environmental variations.

Fungal entomopathogens have been studied for over ten decades as pathogens of insects yet without completely uncovering their full potential as effective biocontrol agents [17]. In this situation, there is a growing concern that fungal entomopathogens with potential to be harnessed as biopesticides may be overlooked by focusing mainly on known species [18]. Well-known genera such as *Beauveria* and *Metarhizium* have gained approval by the United States Environmental Protection Agency as well-characterized biological pest control agents [19] with broad-spectrum activity against arthropod hosts. These two fungal genera are the foremost fungi used for the biological control of arthropod pests. The most important species of these genera that are widely applied for biological control of arthropod pests are *M. anisopliae* and *B. bassiana* [20]. However, it is plausible that many other potential fungi that could be effective for the biocontrol of insects remain unexploited even with the likelihood of obtaining host specific strains [21]. Quinelato and Golo [20] opined that the use of EPF for insect control confers major advantages including finding possible isolates with greater specificity to target hosts due to extended fungal biodiversity, better mode of cuticle penetration, and the environmental safety of the method of application. For example, Hyphomycetes fungi such as the *Aspergillus* species are less isolated and used as biological control agents of arthropods [22,23]. This may perhaps be because it is postulated that opportunistic pathogens can evolve from a saprophytic nature to produce disease in wide range of immunocompromised hosts, although with reduced virulence [24]. *A. flavus* is a fungal pathogen that is well known to be lethal to humans and animals, other than infesting agricultural grains and its ability to secrete aflatoxins [25]. It can cause mild aflatoxicosis which is fatal and or chronic aflatoxicosis which develop into cancers [26]. Because of these features of the fungus, researchers are pessimistic in recommending its application as a biological control agent [25]. However, many studies have found out that not all strains of *A. flavus* are naturally endowed to produce aflatoxins because, in nature, more than a few strains are atoxigenic and cannot produce aflatoxins [27,28,29]. Over the years, atoxigenic strains of *A. flavus* has been widely used for the biocontrol of aflatoxigenic strains in agricultural plantations. These strains are able to control the proliferation of the aflatoxigenic strains in plantations via competitive inhibition and bio-exclusion processes [25,30,31]. This implies that *A. flavus* has the potential to confer a dual advantage when applied as a biological control agent.

Biological control agents such as EPF have been used for the control of seed bugs such as *Diaphorina citri* (Hemiptera: Liviidae) [32], the cotton stainer bug *Dysdercus peruvianus* (Hemiptera: Pyrrhocoridae) [33], the Chagas vector *Meccus pallidipennis* [34], citrus mealybug, *Plannococcus citri* (Hemiptera: Pseudococcidae) [35]. However, there seems to be no studies that have evaluated the potential of EPF for the biological control of *E. pallens* (Hemiptera: Rhyparochromidae).

**Figure 1:**
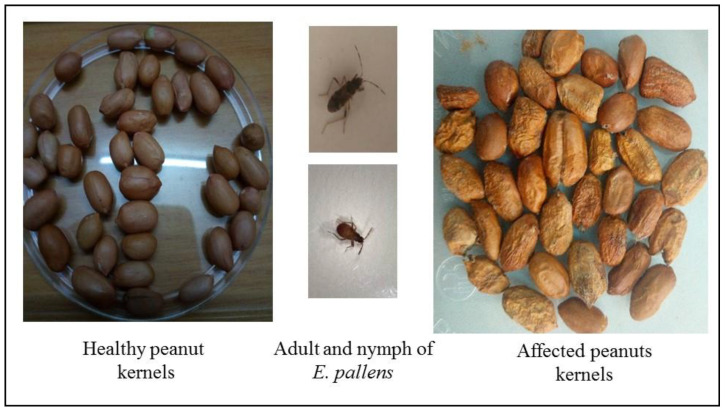
Effect of *E. pallens* attack on peanut kernels. Affected seed kernels become shriveled, losing quality and aesthetic value.

Considering that both adults and nymphs of *E. pallens* attack peanuts, causing both physical damage, loss of seed quality (Figure 1), and the attendant negative effects of synthetic insecticides on the environment, the need for an alternative and environment friendly approach for its control becomes imperative. This suggests the need to explore biological control agents effective for the control of *E. pallens*, other than being restricted to only known species as biocontrol agents. This will be fundamental to knowledge expansion on both the host insect and pathogens. In this study, the potential of EPF against *E. pallens* was evaluated for its biological control under laboratory conditions.

## 2. Materials and Methods

### 2.1. Bugs Collection and Rearing

Adult bugs were collected from groundnut fields in Menglembu, Perak (4°33′58.29″ N and 101°2′53.95″ E), Malaysia, from February to April 2017 in plastic containers and transported to the laboratory. The bugs were maintained in the laboratory as in Khosravi, Sendi [36] with some modifications. The bugs colonies were reared inside plastic cages (40 × 30 × 20 cm) at 25 ± 2 °C and 70 ± 10% relative humidity (RH) under a 14:10 h L:D photoperiods. Cotton balls soaked in sterile distilled water were placed in each container to maintain the humidity within the containers and provide water for the bugs to suck. Fresh groundnut seeds were used as a diet and were changed every 3 days. Gauze materials were placed in each container as a surface for oviposition. Gauze containing oviposited eggs were separated into new containers to hatch at 70 ± 10 (RH). Adult bugs, 2–3 weeks, were used for the virulence bioassay experiments.

#### Identification of Bug Species

The identification of the bug species was done via morphological examinations and molecular method. Genomic DNA from the legs of ethanol-preserved specimens were extracted and used for molecular identification [37], while morphological identification was done according to the method described by Katinka [38].

### 2.2. Fungal Isolates

Cadavers of *E. pallens* were collected from groundnut fields (as mentioned above) inside sterile plastic bags and transported to the laboratory for further analysis. The cadavers were surface sterilized by dipping in 1% (*v/v*) sodium hypochlorite (NaClO) solution for 5 mins and rinsed three times in sterile distilled water. The surface sterilized cadavers were placed on potato dextrose agar (PDA, Oxoid) plate amended with 0.5 g/L chloramphenicol and incubated in the dark at 25 °C for 3 days. Mycelia that developed were sub-cultured until pure cultures were obtained and kept on PDA slants at 4 °C until required. *M. anisopliae* was obtained from the stock culture collection of the Department of Microbiology, University of Ibadan (Nigeria) and used as a standard strain in this study. 

### 2.3. Microscopic Observation

The morphology of the isolates was observed following the conventional slide culture technique. Sterile PDA was cut into small squares of (approximately 1 cm) and a block was placed on a sterile glass slide inside Petri plates (100 × 15 mm) underlaid with sterile filter paper (Whatman 1). Each agar block was inoculated with a fungal colony on the four corners using sterile needles and a coverslip aseptically placed over it. The filter paper underlay was wetted with distilled water and incubated in the dark at 25 °C for 3 days. The coverslip was gently taken off and placed on a glass slide containing a drop of lactophenol cotton blue (LPCB) and examined under the light microscope (Leica DM500, Leica Microsystems, Wetzlar, Germany) with a 40× objective lens [39]. 

### 2.4. In Vitro Screening of Isolates for Aflatoxins Production

Putative *A. flavus* isolated from the bug cadavers were screened for their ability to produce aflatoxins on coconut agar medium (CAM). The screening was done according to the method described by Mamo and Shang [29].

### 2.5. Genomic DNA Extraction and Polymerase Chain Reaction (PCR)

Genomic DNA extraction for both bug and fungi were done using the DNeasy Blood and Tissue Kit (Qiagen, Hilden, Germany) following the manufacturer’s protocol. For the bug DNA, PCR was done according to the protocol of Tembe, Shouche [37] using the primer sets LCO1490 (5′-GGTCAACAAATCATAAAGATATTGG-3′) and HCO2198 (5′-TAAACTTCAGGGTGACCAAAAAATCA-3′), while a fragment of the internal transcribed spacer (ITS) region of the fungal genomic DNA was amplified using the universal primer sets ITS1 (5′-TCCGTAGGTGAACCTGCGG-3′) and ITS4 (5′-TCCTCCGCTTATTGATATGC-3′). PCR protocol was conducted as in White and Bruns [40]. Sequences were aligned and compared to existing sequence data on the GenBank database using the Basic Local Alignment Search Tool (NCBI BLAST) [41].

### 2.6. Phylogenetic Analysis

The phylogenetic analysis involving the ITS sequence data of the isolates was done by comparing with registered sequences on the GenBank database. ITS sequences were aligned, and unnecessary portions removed before the tree was generated using MEGA 7.0 software, which gave the topology and length of the branches [42]. The phylogenetic relationship and the neighbor joining analysis were determined using the Kimura 2-parameter (K2P) model of base substitution in MEGA 7.0 [37]. 

### 2.7. Fungal Conidial Preparation

Fungal isolates were cultured on PDA (Oxoid Ltd., Basingstoke, UK) for 14 days at 25 ± 1 °C under 14:10 h photoperiods. Conidia were harvested by scraping the surface of the medium using sterile wooden spatula into 20 mL 0.05% (*v/v*) Tween 80 (polyoxyethylene sorbitan monooleate, Sigma Chemical Co., St. Louis, MO, USA) inside sterile 50 mL centrifuge tubes and homogenized by vortexing for 3 min. The conidial suspension was sieved through four layers of cheese cloth [20]. A ten-fold serial dilution of the spore suspension was done, and an aliquot estimated on a hemocytometer followed by a final adjustment to 10^8^. For the multiple-dose bioassay, five different conidial concentrations (1 × 10^4^ to 1 × 10^8^ conidia mL^−1^), were prepared.

### 2.8. Conidial Viability

The conidial viability for all isolates was determined by plating 100 µL of 10^6^ conidia mL^−1^ on three replicate plates of PDA. The plates were incubated at 25 °C under 14:10 L:D regimes for 16–24 h. Germination was estimated by counting 300 conidia (100 conidia per area) under a brightfield microscope at 40× magnification. The formation of germ tubes at least half the size of the conidia, was indicative of viability [43].

#### 2.8.1. Single-Dose Virulence Assessment Bioassay

Fungal isolates obtained from cadavers of the bugs and *M. anisopliae* were evaluated for virulence against *E. pallens* according to the method of Orduño-Cruz, Guzmán-Franco [32] with some modifications. Mixed sex adult bugs were used in five replicate groups of 10 each by dipping into 1 × 10^8^ mL^−1^ of conidia for 10 s while control groups were treated with 0.05% (*v/v*) Tween 80. Treated bugs were placed in plastic containers (65 × 45 mm) containing damp sterile filter paper (Whatman 1). Cotton wool soaked in sterile distilled water was placed in each container to provide water for the insect to suck. Afterwards, the treated bugs and the controls were introduced into plastic containers with perforated lids to prevent the insects from escaping, provide ventilation and maintain the required humidity. Both the treatment and the controls were maintained at 25 ± 2 °C, 80 ± 10% relative humidity (RH) and 14:10 h photoperiod without diet. Mortality was scored after every 24 h for 10 days. 

#### 2.8.2. Multiple-Dose Virulence Bioassay

The virulence bioassay of entomopathogenic fungi against *E. pallens* was performed following the method of Resquín-Romero and Garrido-Jurado [44] with some modifications. *A. flavus* and *M. anisopliae,* which produced the highest mortality rates, were selected for the multiple-dose virulence bioassay. Five conidial concentrations were prepared, and the treated bugs and controls were maintained as above. Cadavers were dipped in 1% (*v/v*) sodium hypochlorite (NaClO) solution for 5 min to surface sterilize and rinsed 3 times in sterile distilled water. The disinfected cadavers were incubated at 25 ± 2 °C on sterile wet filter paper in Petri dishes for 5–7 days to activate conidial growth followed by examination under light microscope. 

### 2.9. Data Analysis

Mortality data were corrected using Abbott’s formula [45]. Statistical analyses of data were done using the IBM SPSS 21.0 software (USA). Analysis of conidial viability and bug mortality data was performed using one-way analysis of variance (ANOVA) and the means compared using the least significant difference (LSD) test. Probit analysis was used to estimate the lethal concentration (LC_50_ and LC_90_) and the estimated lethal time (LT_50_ and LC_90_) from infection-confirmed mortalities [46]. All statistical tests were significant at (α) 0.05.

## 3. Results

### 3.1. Molecular and Morphological Identification of E. Pallens

The cytochrome oxidase I (COI) sequence of the bug sample was obtained and used for the molecular identification. The aligned sequence had a COI sequence data of 667 bp after incorporation into GenBank sequence data, thus representing members of the Rhyparochromidae as revealed by congeneric sequences obtained after BLAST. Morphologically, the bug is dark brown in color with small head in relation to the body, a long antenna (four segments) and a rostrum positioned on the head. The bug has two compound eyes, the maxillae and mandibles are fused into a needle-like stylet. Each pair of legs is positioned on a separate thorax and a well-established pronotum. The front wing pairs are positioned on the mesothorax while the metathorax bears the second pairs of wings while the abdomen contains 9 segments. BLAST confirmed the identity of the bug as *E. pallens* (GenBank accession number- MK024388) with 99% similarity with existing sequences on the GenBank database.

### 3.2. Fungal Isolation and Identification

A total of 32 fungi were isolated from the cadavers of *E. pallens* and morphologically identified as *Aspergillus flavus* (11)*, A.niger* (8), *Fusarium proliferatum* (6), *A. tamarii* (4), and *Trichoderma atroviride* (3). The ITS1-5.8S-ITS4 rDNA sequences of representative isolates selected based on colonial growth and conidial viability were sequenced using molecular method. BLAST confirmed the isolates as *Fusarium proliferatum* (F1), *A. tamarii* (F2), *A. flavus* (F3), *Trichoderma atroviride* (F4), and *A. niger* (F5). PCR amplified ITS sequences compared with existing sequence data on the GenBank database gave high level of similarities (Table 1).

### 3.3. In Vitro Screening of Isolates for Aflatoxins Production

**Figure 2:**
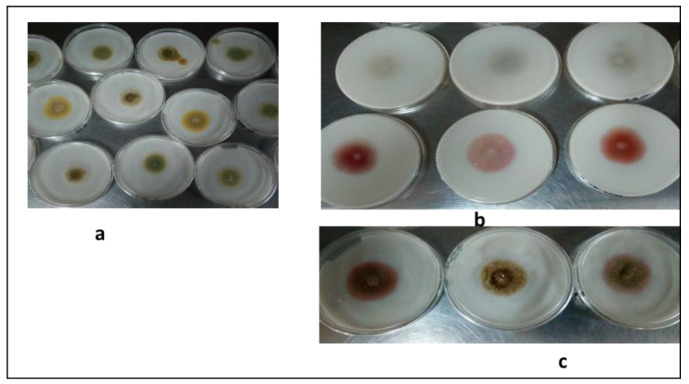
In vitro screening of *A. flavus* isolates for aflatoxins production. (**a**) Growth of *A. flavus* isolates on CAM after 3 days of culture; (**b**) positive isolates (pinkish) and negative isolates (colorless) from the bottom of the plates; (**c**) positive isolates view from the top of the plates.

In the in vitro screening, positive plates containing aflatoxigenic strains appeared pinkish while negative plates with atoxigenic strains appeared colorless (Figure 2). Out of 11 putative isolates of *A. flavus* screened on CAM, only 3 were aflatoxigenic while the remaining 8 were atoxigenic.

### 3.4. Phylogeny of the Fungal Isolates

**Figure 3:**
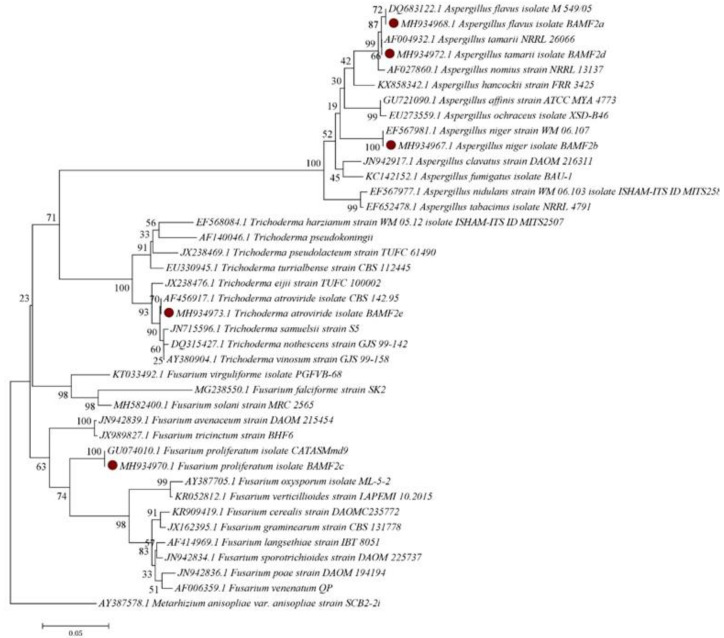
The phylogenetic tree of species-representative fungal isolates internal transcribed spacer (ITS1-5.8S-ITS4) gene sequences constructed using neighbor-joining method. The tree shows genetic relationships between isolates obtained from the cadavers of *E. pallens*. Bootstrap values shown by the nodes are based on 1000 replicates. Red dots show isolates used in this study.

The MEGA 7.0 software was used to construct the phylogenetic tree based on the sequence data of the ITS1-5.8S-ITS4 rDNA region. The fungal ITS sequence data were used for species identification and construction of phylogenetic relationship. The phylogenetic analysis showed that the 5 fungal genera had high level of similarities to existing sequences previously reported on the GenBank database (Figure 3). However, the *M. anisopliae* used in this study was isolated from *Zonocerus variegatus* cadavers. Isolates with ≥90% conidial viability (Table 2) were selected for the in vitro screening and bioassay against *E. pallens*.

### 3.5. Single-Dose Virulence Assessment Bioassay

**Figure 4:**
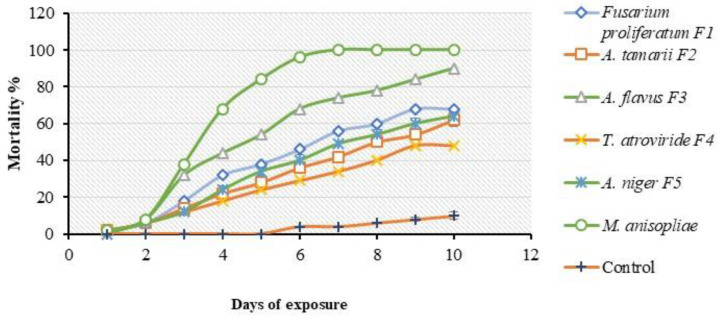
Virulence of six isolates of EPF against *E. pallens* treated with 1 × 10^8^ conidia mL^−1^. Mortality of the bugs was observed after every 24 h for 10 days post-treatment.

All fungal isolates tested in the bioassay demonstrated the capacity to infect the bugs. The fungal isolates produced different mortality rates against *E. pallens* and were found to be significantly different (*F*_6,70_ = 5.758; *p* < 0.0001). At 7 days after treatment, *M. anisopliae* caused 100% mortality of the bug after exposure while *A. flavus* (F3) caused 90% cumulative mortality of the bugs 10 days after treatment, followed by *F. proliferatum* (F1) 68%, *A. niger* (F5) 64%, *A. tamarii* (F2) 62%, and *T. atroviride* (F4) 48% compared to the control where mortality never reached 15% (Figure 4). *A. flavus* (F3) and *M. anisopliae* produced the highest mortalities and were selected for the multiple-dose virulence bioassay to further determine their virulence potential.

Other than corrected mortality, the mean mortality of *E. pallens* treated with a single concentration (1 × 10^8^ conidia mL^−1^) was computed for all the fungal isolates tested in the single-dose virulence assessment test. The mean mortalities of the bug were higher between the 4th and 6th days of exposure to the fungal conidia of all isolates (Table 3) compared to the controls. Mortality of the bug due to *A. flavus* (F3) was 90% 10 days after treatment. The mean mortalities of the bugs were significantly different between the days of exposure and individual isolates (*F. proliferatum* (F1): *F_5_*_,12_ = 25.85, *p* < 0.0001; *A. tamarii* (F2): *F*_5,12_ = 14.30, *p* < 0.0001; *A. flavus* (F3): *F*_5,12_ = 164.80, *p* < 0.0001; *T. atroviride* (F4): *F*_5,12_ = 7.70, *p* < 0.002; *A. niger* (F5): *F*_5,12_ = 30.48, *p* < 0.0001). 

### 3.6. Multiple-Dose Virulence Bioassay

**Figure 5:**
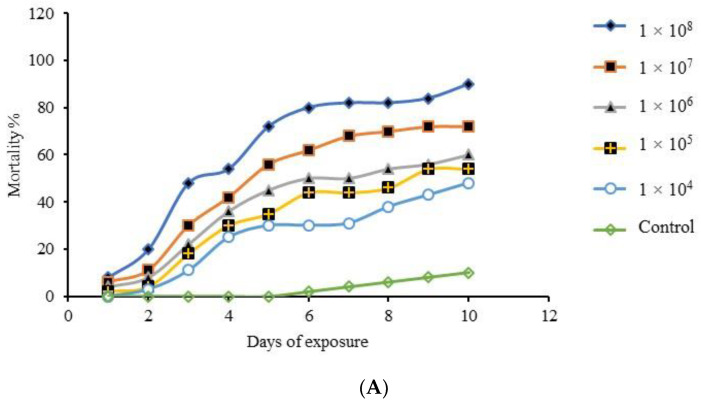

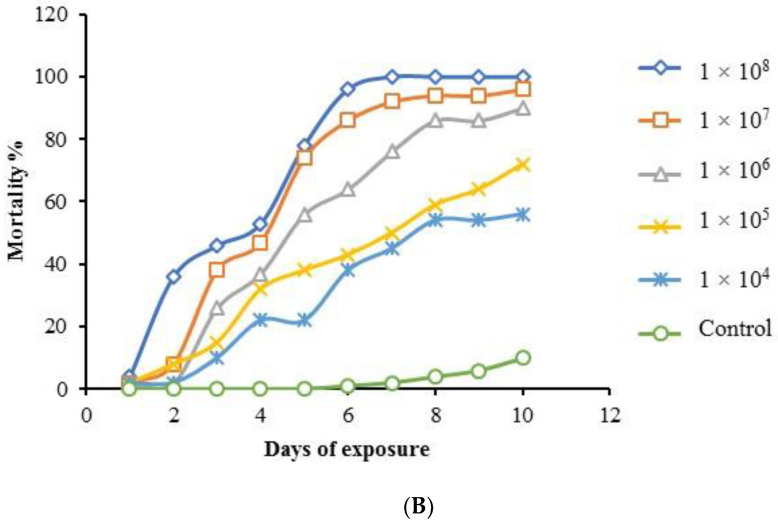
Cumulative mortality of *E. pallens* after treatment with fungal isolates at different conidial concentrations. (**A**) Response of *E. pallens* after immersion into different concentrations of *A. flavus* (F3) conidia; (**B**) response of *E. pallens* exposed to different concentrations of *M. anisopliae* conidia.

The bioassay utilized different conidial concentrations of the EPF which produced mortality rates proportional to increase in the conidial concentrations used. Here, *A. flavus* (F3) produced a significant effect on the mortality of the bugs (*F*_5,6_ *=* 5.644, *p* < 0.0001) with confirmed mortality ranging from 48% at 1 × 10^4^ conidia mL^−1^ to 90% at 1 × 10^8^ conidial mL^−1^ 10 days after treatment (Figure 5A). However, *M. anisopliae* also produced significant mortality of the bugs (*F*_5,60_ *=* 6.493, *p* < 0.0001) where a concentration of 1 × 10^4^ conidia mL^−1^ produced 56% cumulative mortality 10 days after treatment while 1 × 10^8^ conidia mL^−1^ caused a cumulative mortality of 100% 7 days after treatment, respectively, compared to the control (Figure 5B). 

The LC and LT values which are measures of virulence among EPF was determined in terms of conidial concentration and time taken for mortality to occur. The LC_50_ and LC_90_ values obtained were 6.75 × 10^6^ and 4.42 × 10^9^ conidial mL^−1^ for *A. flavus* (F3) compared to 8.0 × 10^6^ and 6.14 × 10^8^ conidial mL^−1^ for *M. anisopliae*, respectively (Table 4), while the LT_50_ and LT_90_ values of the test isolates were 3.3 and 6.2 days for *A. flavus* (F3) and 3.6 and 5.6 days for *M. anisopliae*, respectively (Table 5). 

## 4. Discussion

Entomopathogenic fungi are known natural pathogens infecting insect hosts that can be collected from the field environment either infected or dead and incubated under laboratory conditions to isolate, document, and use the pathogens as biological control agents [56]. In this study, different species of fungi were isolated from the cadavers of *E. pallens*, some of which have been reported in previous studies. For example, the isolation of *A. flavus* from the cadavers of insects have similarly been reported in Lee and Kim [15], Assaf and Haleem [53], and [25], respectively; whereas the isolation of *F. proliferatum* from the asparagus beetle, *Crioceris asparagi*, was reported in [52]. Gardezi [48] reported the isolation of *A. tamarii* from insect cadaver and testing its pathogenicity against several insect species. *A. flavus* was found to be the most abundant isolate among the fungal isolates identified. Similar finding has been reported in a study on the almond bark beetle, *Scolytus amygdali* [57]. However, all the fungal isolates demonstrated pathogenicity against *E. pallens* but at different degrees under the same experimental conditions. This may be attributed to established facts that sucking, forest, and soil-dwelling insect pests are very much susceptible to infection by EPF, because of the vulnerability of their cuticle to conidial adhesion, germination, and penetration contrary to control agents such as bacteria, viruses, parasitoids, and nematodes that must be ingested to initiate infection [58]. Their mechanisms of action are said to be due to inherent pathogenicity (the infective capacity of an entomopathogen resulting in disease) and virulence (the degree to which the host tissues are colonized by the pathogen with time) traits, which are gene-specified intrinsic features of EPF. However, these intrinsic features are largely dependent on host immune response, nature of formulations, growth medium composition, abiotic factors, and methods of application used [59].

Although there is an existing pessimism on the use of *A. flavus* as a biological control agent because of its potential for aflatoxin synthesis, this study confirmed that not all isolates are aflatoxigenic. This was based on the fact that, from the eleven *A. flavus* isolates obtained in this study, 8 (72.7%) were found to be atoxigenic while only 3 (27.3%) were aflatoxigenic (Figure 2). Similarly, Gupta and Gopal [25] observed that out of seven *A. flavus* isolates obtained from three insect groups—*Stephanitis typica* (lace bug), *Opisina arenosella*, and *Proutista moesta*—only two of the isolates were aflatoxigenic. Wicklow and Dowd [60] reported that atoxigenic strains of *A. flavus* were lethal to the maize corn insect *Carpophilus hemipterus* due to their ability to secrete certain sclerotial metabolites. 

The single-dose virulence assessment bioassay shows that all isolates tested were pathogenic against *E. pallens*, although the rate of mortalities differed (*p* < 0.0001). *A. flavus* (F3) and *M. anisopliae* showed greater potential for virulence against *E. pallens*. *A. flavus* (F3) produced a mortality rate of 90% after 10 days compared to *M. anisopliae* which caused 100% mortality of the bugs 7 days after treatment. The killing ability of other fungal isolates were lower compared to the two isolates described (Figure 4). The two most effective entomopathogens were selected for virulence bioassay against the bug based on their lethal effects, which could be related to the assertion by Ferron [61], that both fungal species can secrete lipolytic enzymes during infection, which enables them to degrade the proteo–chitin complex. This may likely explain why the two isolates showed more virulence than the other isolates used. Studies have established that killing of insects by entomopathogenic fungi involves a series of successive steps that could lead to the death of the host depending on its ontogenic stage or immune response. These steps involve: (1) adhesion of the fungal conidia on the integument of the insect; (2) conidial germination under optimum conditions to form germ tubes; (3) degradation of cuticular structures by hydrolytic enzymes and mechanical effect to enable penetration; (4) conversion of the hyphae into blastospores to exploit nutrients in the host hemocoel; (5) blastospores utilize the available sugars and as well release toxins inside the hemolymph; (6) blastospores suppress the host immune system and release toxins that expedite killing of the host; (7) fungus exits the host through openings on the cuticle to produce spores on the cadaver surface [41,62]. Furthermore, Ferron [61] opined that once total invasion of the cadaver occurs, fungal conidiation on the surface of the mummified insect cadaver is dependent on the ambient environmental relative humidity. The mycelia develop from within the cadavers to the surface to produce conidiophores only when the atmosphere becomes saturated. Otherwise, the mummified cadaver remains dry and brittle. Under this condition, entomogenous agents become preserved in the form of chlamydospores.

In the multiple-dose virulence bioassay involving the two isolates (Figure 5), *A. flavus* (F3) induced 90% cumulative mortalities of *E. pallens* in a fashion similar to Seye and Bawin [23] who reported high mortalities of the aphid bug *Acyrthosiphon pisum* caused by *A. flavus* and *A. clavatus* isolates, respectively. Gopal and Gupta [63] also reported 90% mortalities of *Opsina arenosella* larvae after 5 days when exposed to *A. flavus* AF2 (ITCC 5005) at a concentration of 10^6^ conidia mL^−1^. Similarly, Gupta and Gopal [25] reported 80% mortalities of nymphs of *Stephanitis typica* within 3 days of exposure to *A. flavus* (ITCC 5004). Generalist facultative EPF such as *B. bassiana*, *M. flavoviride*, and *Paecilomyces* spp cause a sigmoidal dose-mortality curve during the killing of susceptible hosts [64]. But Scully and Bidochka [24] evaluated two strains of the opportunistic pathogens *A. flavus* 9308 and *A. flavus* 6982 against *Galleria mellonella* larvae and found that the isolates demonstrated low virulence regardless of the dose used, such that instead of a sigmoidal curve, a rather flat curve was obtained. The study reported that no significant difference was observed with further increase in conidial concentration. However, Karthi and Vaideki [22] reported 71% and 63% mortalities of fourth and third instars of *Spodoptera litura* exposed to *A. flavus* at the highest conidial concentration of 4 × 10^6^ conidia mL^−1^. 

However, for bugs treated with *M. anisopliae*, mortalities in *E. pallens* reached 100% after 7 days of treatment. Similarly, Loureiro and Moino-Jr [65] observed a 100% cumulative mortalities of the aphid (Hemiptera), *Aphis gossypii*, and *Myzus persicae* after 7 days of treatment with *M. anisopliae*. Also, a 100% cumulative mortality rate of the aphid *Aphis craccivora* treated with 10^8^ conidial mL^−1^ of *M. anisopliae* was earlier observed by Saranya and Ushakumari [66] after 4 days of exposure to the fungus. Furthermore, Santos and Freitas [59] reported 100% cumulative mortality of the Hemiptera *Thaumastocoris peregrinus* exposed to 10^8^ conidia mL^−1^ of *M. anisopliae* after 10 days. The differences between these findings may be attributed to factors such as isolates and strains variations, types of insect species, host immune responses, and the prevailing environmental conditions. 

The LC and LT as yardsticks that measure virulence in terms of conidial concentrations and time length required to kill the test insect population exposed to a given concentration of a pathogen were determined. In the multiple-dose virulence bioassay against *E. pallens*, *A. flavus* (F3) showed lower median lethal concentrations (LC_50_) and median lethal time (LT_50_) compared to that of *M. anisopliae* (Table 3 and Table 4). This shows that the isolate *A. flavus* (F3) was more virulent at killing 50% of the test *E. pallens* population faster and at lower conidial concentration compared to *M. anisopliae*. However, on the contrary, *M. anisopliae* progressed to achieve a lower LC_90_ and LT_90_ compared to *A. flavus* (F3) (Table 3 and Table 4). This could be attributed to the degree of persistence of the pathogens within the host hemocoel, nutrient exhaustion in the host, and the host’s immune response to the pathogens over time. The ability of *M. anisopliae* to persist against the host’s immune responses, exploit available nutrients, and withstand ambient environmental changes would have been responsible for the rise in virulence over *A. flavus* (F3) at the later stage of the bug infection. Seye and Bawin [23] had earlier reported higher virulence for *A. flavus* isolate against the aphid *Acyrthosiphon pisum* (Hemiptera: Aphididae) after 5 days of treatment, producing a lower LC_50_ and LC_90_ values (1.23 × 10^3^ and 1.34 × 10^7^ conidia mL^−1^) compared to *M. anisopliae* (3.67 × 10^3^ and 9.71 × 10^7^ conidia mL^−1^). Karthi and Shivakumar [22] did report higher virulence of *A. flavus* isolates against 3rd instar and 4th instar nymphs of *Spodoptera litura*, respectively. However, the observations in this study shows that *A. flavus* (F3) possess the capacity to perform better in virulence against some arthropod pest than *M. anisopliae*, though its insecticidal activities are rarely reported compared to the widely discussed *M. anisopliae*. But some studies have reported LC_50_ values lower than what was obtained in this study. For example, FitzGerald and Hill [35] observed a lower LC_50_ (5.29 × 10^5^ conidia mL^−1^) for *M. anisopliae* compared to what was found in this study. Also, Ekesi and Akpa [67] described how four different strains of *M. anisopliae* differed in virulence against the aphid *Aphis craccivora* (Koch), with LC_50_ values ranging between 3.1 × 10^5^ to 7.4 × 10^6^ conidia mL^−1^. But Shah and Wang [68] asserted that fungal species and strains virulence, substrate composition, and culture methods determine to a large extent whether insecticidal compounds responsible for virulence are produced by conidia of EPF. 

The lethal time (LT_50_ and LT_90_) obtained for both fungi used in this study agrees with the finding of Mweke et al. (2018) who observed 3.3 to 6.3 days for EPF tested against *Aphis craccivora*. But Saranya and Ushakumari [66] reported an LT_50_ of 5.54 days for *M. anisopliae* against the *Aphis craccivora*, which is longer than obtained in this study. This therefore means that the isolates used in this study showed virulence potential against *E. pallens*, probably due to the host type, method of fungal conidial application, and the virulence of the strains used. 

Death of the insects due to fungal parasitism was confirmed when the insect body becomes mummified and brittle. However, to validate whether death was due to infection by the fungal pathogens, the cadavers were cultured and examined for the development of fungal conidia. This is important such that where these fungal agents are applied as mycopesticides, they must confer the relative advantage of producing spores on the host cadavers to serve as a secondary source of inoculum for continuous propagation, natural host infection, reduction of insect menace, and costs of applications [69]. Since *E. pallens* attack peanuts by piercing the pods with their rostrum when they congregate under harvested groundnut on the field, secondary infection by EPF which is largely dependent on insect behavior becomes easier due to their susceptibility under such conditions. Treating this bug species with EPF exposes them to infection by the pathogens due to their social interactions and susceptibility of their cuticles under appropriate environmental conditions.

## 5. Conclusions

In this study, *A. flavus* was found to be more virulent against *E. pallens* compared to *M. anisopliae* based on their LC_50_ and LT_50_ values—killing half the population of the test bugs with lower conidial concentrations and at a reduced exposure time. Although it has often been less applied for the biological control of insect pests compared to the widely used *M. anisopliae*, yet, it has potential for the biological control of *E. pallens* and should be considered in myco-formulations for field applications. Further research on the field evaluation and development of these EPF into biocontrol agents of *E. pallens* and their incorporation into integrated pest management (IPM) systems should be explored.

## Figures and Tables

**Table 1:** Morphological features and identification of fungi isolated from the cadavers of *E. pallens.*

Isolates	Growth Morphology	Colony Color	Phialides Organization	Conidia Shape	Conidia Size (µm)	Probable Fungus	Strain Number	Reference	Molecular Identity	Identity %	Accession No.
F1	Luxuriant mycelium which becomes folded as culture ages	Peach with purple trace, whitish spots	Simple lateral	Oval, cylindrical, ellipsoidal to macro and microconidia	3.8–6.6 × 1.5–3.4	*Fusarium proliferatum*	BAMF2c	[47]	*Fusarium proliferatum*	100	GQ505459.1
F2	Fast growing	Green	Biseriate and radiate	Rough and globose	3–5.5 × 2.4–2.9	*Aspergillus tamarii*	BAMF2d	[48]	*Aspergillus tamarii*	99	LC127424.1
F3	Rapid growth and densely sporulated	Dirty green	Naturally radiate	Classically globose to subglobose	3.5–5.2 × 2.2–2.8	*A. flavus*	BAMF2a	[49]	*A. flavus*	100	MF319893.1
F4	Rapid growth, greyish	Glaucous to dark green	Verticillate, solitary, lageniform, and curved	Smooth, dark green, sub globose when fully mature	35–7 × 2.5–3.5	*Trichoderma atroviride*	BAMF2e	[50]	*Trichoderma atroviride*	100	KU896311.1
F5	Rapid growth and conidiation	Black	Biseriate	Coarse, echinulate and globose	4–6 × 2.7–3.8	*A. niger*	BAMF2b	[49]	*A. niger*	100	KY657577.1
Ref.	Rapid growth and conidiation	Dark-herbage green	Cylindrical and podgy	Colorless, Ellipsoidal, rounded apex, slightly truncate base	4.8–6.1 × 2.2–3.6	*M. anisopliae*		[41,51]	*M. anisopliae*		

**Table 2:** Occurrence and conidial viability of the isolates.

Isolate	Name of Species	Number of Isolates	Mean Conidial Viability (%) ± S.E	Reports of Pathogenicity Against Insects
F1	*Fusarium proliferatu*	6	94 ± 0.33a	[52]
F2	*A. tamarii*	4	93 ± 0.88a	[48]
F3	*A. flavus*	11	97 ± 0.88a	[15,22,49,53]
F4	*Trichoderma atroviride*	3	90 ± 0.33a	Mostly used for the competitive exclusion of plant pathogens.
F5	*A. niger*	8	95 ± 0.33a	[49,54]
Ref	M. anisopliae *		96 ± 0.33a	[32,34,49,55]

Key: * = is from cadaver of *Zonocerus variegatus* [49], while other isolates were obtained from an *E. pallens* cadaver. Percent conidial germination ± standard errors. Means followed by the same letter in a column are not significantly different (*p* > 0.05) according to the least significant difference (LSD) test.

**Table 3:** Mortality of *E. pallens* treated with 10^8^ conidial mL^−1^ of fungal isolates.

Isolate	Name of Species	Mean Mortality ± S.E					
		Day 2	Day 4	Day 6	Day 8	Day 10	Control
F1	*Fusarium proliferatum*	3.33 ± 0.88	15.67 ± 1.76	8.33 ± 0.67	9.67 ± 1.45	2.67 ± 0.33	3.34 ± 0.15
F2	*A. tamarii*	2.00 ± 2.08	13.33 ± 1.86	12.33 ± 1.20	6.67 ± 2.67	2.67 ± 0.67	2.33 ± 0.33
F3	*A. flavus*	3.67 ± 1.76	25.67 ± 0.33	10.00 ± 0.58	1.33 ± 0.88	0.00 ± 0.00	1.67 ± 0.33
F4	*T. atroviride*	3.67 ± 1.67	10.67 ± 1.33	10.00 ± 1.53	8.00 ± 1.15	5.33 ± 0.88	2.33 ± 0.33
F5	*A. niger*	2.33 ± 1.20	15.00 ± 2.31	11.00 ± 0.58	6.00 ± 1.53	2.00 ± 1.16	2.67 ± 0.56
Ref.	*M. anisopliae*	4.00 ± 0.58	29.33 ± 1.76	16.30 ± 1.86	1.37 ± 0.00	0.00 ± 0.00	3.86 ± 0.78

*F. proliferatum*: *F_5_*_,12_ = 25.85, *p* < 0.0001; *A. tamarii*: *F*_5,12_ = 14.30, *p* < 0.0001; *A. flavus*: *F*_5,12_ = 164.80, *p* < 0.0001; *T. atroviride*: *F*_5,12_ = 7.70, *p* < 0.002; *A. niger*: *F*_5,12_ = 30.48, *p* < 0.0001. Conidial concentration = 1.0 × 10^8^ conidia mL^−1.^

**Table 4:** Probit analysis results (LC_50_ and LC_90_ expressed as conidia mL^−1^) for the virulence of *A. flavus* (F3) and *M. anisopliae* against *E. pallens* (Hemiptera: Rhyparochromidae).

Isolate	LC_50_Conidia/mL	95% Fiducial Limits		LC_90_	95% Fiducial Limits	
		Lower	Upper		Lower	Upper
***A. flavus* F3**	**6.75 × 10^6^**	1.38 × 10^6^	4.11 × 10^7^	4.42 × 10^9^	1.01 × 10^9^	2.57 × 10^12^
*M. anisopliae*	8.0 × 10^6^	1.41 × 10^6^	1.2 × 10^7^	6.14 × 10^8^	2.54 × 10^8^	1.76 × 10^9^

**Table 5:** Summary of probit analysis on lethal time (LT) of *A. flavus* (F3) and *M. anisopliae* against *E. pallens* treated by dipping into conidia of the fungi.

Isolate	LT_50_ (Days)	95% Fiducial Limits		LT_90_ (Days)	95% Fiducial Limits	
		Lower	Upper		Lower	Upper
***A. flavus* F3**	**3.3**	1.3	4.6	6.2	5.9	7.1
*M. anisopliae*	3.6	1.6	4.7	5.6	5.3	7.3
